# First report of *Angiostrongylus vasorum* parasitizing lesser grison (*Galictis cuja*) (Mustelidae) in Brazil

**DOI:** 10.1007/s11259-026-11418-7

**Published:** 2026-07-24

**Authors:** Julia Somavilla Lignon, Maiza Paixão Souza, Natália Büttenbender, Pedro Almeida Rezende, Raqueli Teresinha França, Kauê Rodriguez Martins, Rodrigo Casquero Cunha, Felipe Geraldo Pappen, Diego Moscarelli Pinto

**Affiliations:** 1 Laboratório do Grupo de Estudos em Enfermidades Parasitárias, Departamento de Veterinária Preventiva, Universidade Federal de Pelotas, Pelotas, Rio Grande do Sul Brasil; 2 Núcleo de Reabilitação da Fauna Silvestre, Universidade Federal de Pelotas, Pelotas, Rio Grande do Sul Brasil; 3 Laboratório de Biologia Molecular Veterinária, Departamento de Veterinária Preventiva, Universidade Federal de Pelotas, Pelotas, Rio Grande do Sul Brasil

**Keywords:** Angiostrongylosis, Nematoda, Wild fauna, Rio Grande do Sul

## Abstract

*Angiostrongylus vasorum* is a metastrongylid nematode that parasitizes the pulmonary arteries and right heart of canids, being the etiological agent of canine angiostrongylosis, a disease of growing veterinary concern due to its expanding geographic distribution and the involvement of domestic and wild hosts. In Brazil, the parasite has been reported mainly in domestic dogs and some wild canids, and its occurrence in mustelids has been documented only once previously. This study reports the first occurrence of *A. vasorum* infecting a lesser grison (*Galictis cuja*) in Brazil. The mustelid was found after being accidentally run over by a vehicle on a highway in the south of the country. Clinical, physical, and complementary examinations were performed for clinical screening. In the parasitological examination of feces, nematode larvae were recovered and identified morphometrically and molecularly through conventional PCR targeting to the *COX1* gene, followed by sequencing and phylogenetic analysis. The results confirmed the presence of *A. vasorum*, showing 99–100% identity with sequences of the same species deposited in GenBank. Therefore, we document here the first report of *A. vasorum* parasitizing *G. cuja*. Thus, the number of known host species parasitized by this helminth is expanded. Furthermore, we provide a new molecular sequence, contributing to the knowledge of this nematode in South America.

## Background

*Angiostrongylus vasorum* (Baillet 1866) (Nematoda: Metastrongylidae) is a nematode considered a parasite of growing importance in veterinary medicine (Di Cesare and Traversa 2014). It primarily affects domestic and wild canids, although it may also parasitize other carnivores (Tayyrov et al. 2021). Commonly known as the “French heartworm,” it is the etiological agent of canine angiostrongylosis, a potentially severe disease that mainly affects the respiratory and cardiovascular systems of the hosts, ranging from asymptomatic infections to severe and occasionally fatal clinical manifestations (Ferdushy and Hasan 2010; Rinaldi et al. 2014). The clinical presentation is associated with inflammation triggered by the presence of adult parasites, egg deposition, and migration of first stage larvae (L_1_) within the pulmonary artery and its branches, as well as in the right ventricle of the heart (Di Cesare and Traversa 2014). The life cycle of *A. vasorum* is indirect and is maintained predominantly by terrestrial gastropods as intermediate hosts, in which the larvae develop to the infective third stage (L_3_). Experimental studies have demonstrated that some freshwater gastropods, including *Biomphalaria glabrata* (Say 1818) and *Pomacea canaliculate* (Lamarck 1822), are susceptible to infection and can support larval development to the infective L_3_ stage under laboratory conditions (Barçante et al. 2003; Mozzer et al. 2015). Definitive hosts become infected through ingestion of infected gastropods or, less frequently, paratenic hosts carrying infective larvae (Di Cesare and Traversa 2014).

The geographic distribution of the parasite includes many countries in Europe, North and South America, as well as Africa (Ferdushy and Hasan 2010). Although the parasite has been reported in domestic and wild canids from different Latin American countries, suggesting an endemic presence throughout the continent, few 2009epidemiological surveys have been conducted in recent decades, indicating that this parasitosis remains both underestimated and neglected (Penagos-Tabares et al. 2018). Furthermore, important gaps remain regarding the role of wild hosts in the maintenance and dissemination of the parasite, especially in South America, where available data are scarce and fragmented. The red fox (*Vulpes vulpes* Linnaeus 1758), a species widely distributed in Europe and North America, is recognized as the main natural definitive host of the *A. vasorum*, playing an important role in maintaining the cycle and in transmission to domestic animals (Schnyder et al. 2010; Taulescu et al. 2023; Rojas et al. 2024).

Over the last decades, *A. vasorum* has shown a significant geographic expansion and is currently considered an emerging parasite in several regions worldwide (McGarry and Morgan 2009; Di Cesare and Traversa 2014; Taulescu et al. 2023). This scenario reinforces the importance of studies involving wild hosts, particularly in areas where epidemiological information remains limited, such as Brazil. In mustelids, reports of *A. vasorum* are rare and sporadic, having been described only in the European otter (*Lutra lutra* Linnaeus 1758) in Denmark (Madsen et al. 1999), badger (*Meles meles* Linnaeus 1758) in Spain (Torres et al. 2001), tayra (*Eira barbara* Linnaeus 1758) in Brazil (Vieira et al. 2008) and weasel in England (*Mustela erminea* Linnaeus 1758) (Simpson et al. 2016). Studies involving *Galictis cuja* (Molina 1782) are even scarcer, particularly regarding its helminth fauna and the potential epidemiological role of this species in maintaining parasites of veterinary importance.

Parasitological surveillance in wildlife represents a fundamental tool for understanding the transmission dynamics of emerging helminths, allowing the identification of potential natural reservoirs and routes of dissemination between wild and anthropized environments. In this context, the lack of available information reinforces the need for investigations on the diversity and distribution of helminths in different hosts and geographic regions. In the present study, we report for the first time the occurrence of *A. vasorum* in *G. cuja*.

## Case presentation

An adult female lesser grison (*G. cuja*), was found after being accidentally struck by a vehicle on a highway on BR 293 in Pinheiro Machado (31°33’49.3"S 53°23’45.9"W), Rio Grande do Sul (RS) and was taken to the Núcleo de Reabilitação da Fauna Silvestre (NURFS) at the Federal University of Pelotas. During the initial clinical examination, the animal presented findings consistent with mild traumatic brain injury resulting from the collision, including hematomas in the oral cavity and upper eyelid, as well as motor incoordination. No ectoparasites were observed. A specific clinical examination revealed no abnormalities in the cardiorespiratory, musculoskeletal, integumentary, or urogenital systems, and the animal was released after the clinical treatment period. Complementary tests, including a complete blood count and parasitological fecal examination, were requested as part of the routine clinical screening protocol for wild animals admitted to the rehabilitation center. No abnormalities were detected in the hematological analysis.

For the parasitological fecal examination, three fecal samples were collected on consecutive days from the enclosure using gloves and sterile collection containers immediately after defecation. The samples were transferred in an insulated cooler containing ice to the laboratory of the Grupo de Estudos em Enfermidades Parasitárias (GEEP) at the Federal University of Pelotas and kept refrigerated at 4 °C until analysis. The following parasitological techniques were performed: modified zinc sulfate centrifugal flotation (Monteiro 2017), spontaneous sedimentation (Hoffman et al. 1934), and Baermann-Moraes technique (Moraes 1948). For parasite identification, all diagnostic morphological characteristics observed during the parasitological examination were evaluated to allow identification at the lowest possible taxonomic level. These included, when applicable, eggshell morphology and ornamentation, embryonic and larval development, and the presence of opercula and aculei. Identification was performed by comparing the observed morphometric characteristics with published descriptions of helminth eggs, considering the known parasite fauna of the host species (Corrêa et al. 2016; Soares et al. 2018), using an Olympus optical microscope (CX22 series) (Olympus Corporation, Tokyo, Japan). Eggs belonging to the family Trichostrongylidae, eggs of *Capillaria* spp., and first-stage nematode larvae (L_1_) were observed.

Five L_1_ larvae were recovered from the feces and morphometrically identified according to the descriptions provided by McGarry and Morgan (2009), Rinaldi et al. (2014), Penagos-Tabares et al. (2018) and Taulescu et al. (2023). The mean and standard deviation were calculated based on measurements of the five larvae using the EpiTools epidemiological calculator (Sergeant 2018).

**Fig. 1:**
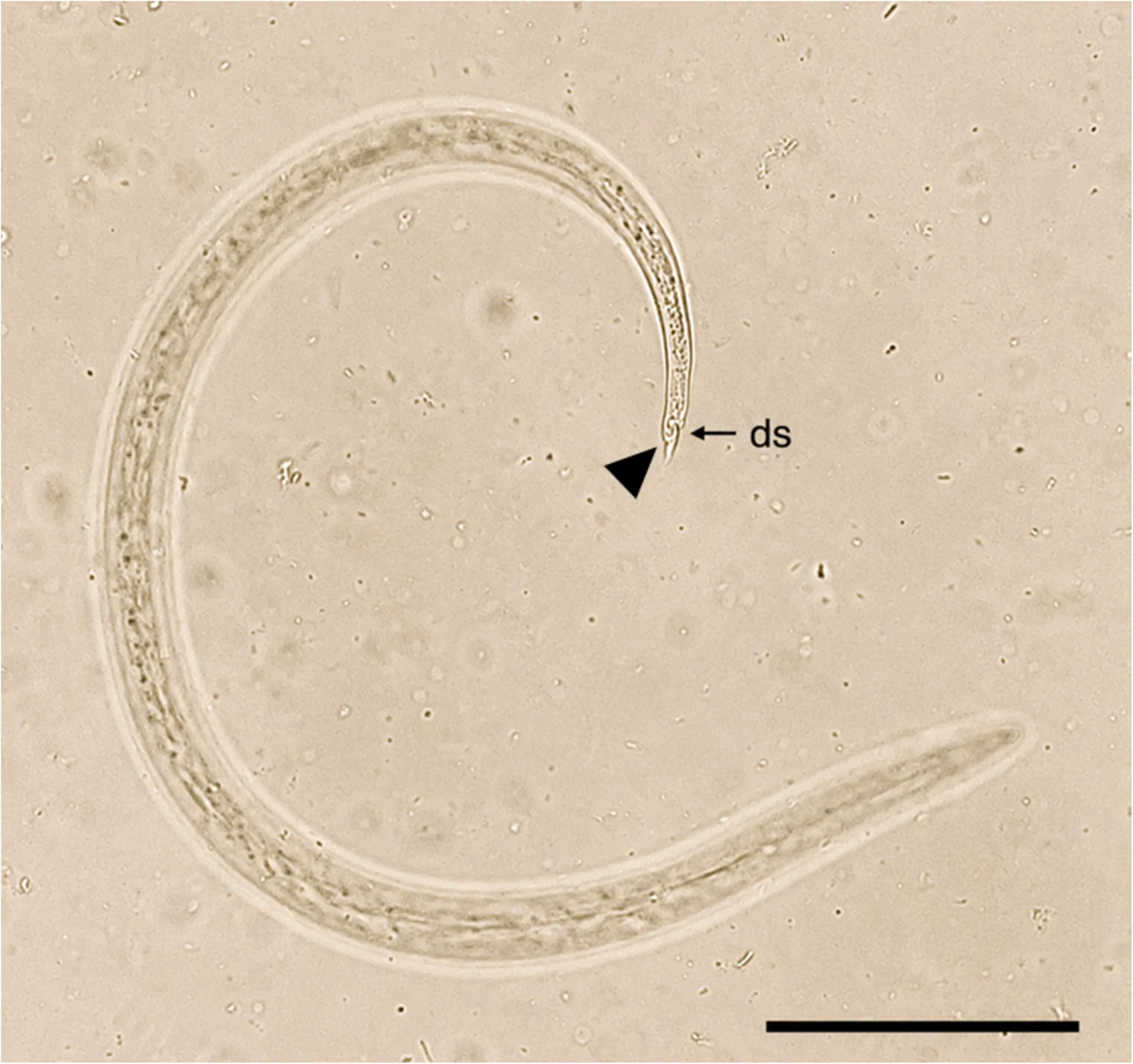
First-stage larva (L1) of *Angiostrongylus vasorum* showing a dorsal spine (ds) and ventral indentation (arrowhead), recovered from the feces of a *Galictis cuja*. Scale bar = 60 μm

The L_1_ larvae exhibited morphometric characteristics and morphology of the caudal extremity (Fig. 1), with a sinusoidal curvature, a dorsal spine, and a ventral indentation (McGarry and Morgan 2009; Penagos-Tabares et al. 2018; Taulescu et al. 2023) consistent with *A. vasorum.* The L_1_ larvae measured 399.6 (± 1.34) µm in length and 14 (± 0.70) µm in width, with a non-rhabditiform esophagus extending to approximately one-third of the total larval length and the presence of a small button-shaped cephalic knob emerging from the oral extremity (Penagos-Tabares et al. 2018). The larvae were photographed using an Olympus optical microscope (CX22 series) (Olympus Corporation, Tokyo, Japan) equipped with a digital image capture system.

The larval DNA was extracted from a pool of 10 L_1_ larvae using the Quick-Zol (Trizol) reagent (Ludwig Biotechnology, Porto Alegre, Rio Grande do Sul, Brazil) following the manufacturer’s instructions. DNA samples were quantified using a ultraviolet light spectrophotometer (Thermo Scientific NanoDrop Lite Spectrophotometer, Waltham, Massachusetts, USA) to assess their quality by measuring their purity (A260/A280 ratio), with samples ranging from 1.8 to 2.2, and concentration in nanograms per microliter (109.3ng/µL), which were stored at −20 °C until PCR was performed. Additionally, 1% agarose gel electrophoresis was conducted to confirm the integrity of the extracted material.

A conventional Polymerase Chain Reaction (PCR) was performed using the following primers: JB3 (5’ TTTTTTGGGCATCCTGAGGTTTAT 3’) and JB4.5 (5’ TAAAGAAAGAACATAATGAAAATG 3’) (Bowles and McManus 1994), amplifying a fragment of approximately 420 base pairs (bp) of the mitochondrial gene subunit I of cytochrome *c* oxidase (*COX1*) according. In the reaction, 2.0 µL of DNA (50 ng/µL) and the mixture containing 2.0 µL of dNTP (2.5 mM), 1.0 µL of each primer (10 µM), 2.5 µL of buffer solution (10X), 1.25 µL of MgCl2 (50 mM), 0.25 µL of Taq DNA polymerase (5U/µL), and 15 µL of ultrapure water were used, totaling 25 µL. The amplifications in a conventional thermocycler included: initial denaturation at 94 °C for 2 min, followed by 35 cycles at 95 °C for 1 min, 50 °C for 1 min, 72 °C for 1 min, final extension at 72 °C for 10 min, followed by a hold at 4 °C. DNA from adult nematodes of *Toxocara canis* was used as a positive control. Ultrapure water was used as a negative control. The amplified products were analyzed by 1.5% agarose gel electrophoresis, stained with ethidium bromide (0.5 µg/mL), and visualized under ultraviolet light. A 100 bp molecular weight marker (Ladder 100 bp 500 µl, Ludwig Biotechnology, Alvorada, RS, Brazil) was used.

The amplicons were excised and purified using a Gel Purification Kit (Ludwig Biotechnology, Alvorada, RS, Brazil), according to the manufacturer’s recommendations, and then subjected to sequencing using the BigDye Terminator Cycle Sequencing Kit v3.1 (Thermo Fisher, USA) on an ABI3500 genetic analyzer (Applied Biosystems, USA). Consensus sequences were obtained by electrogram analysis with Phred base calling and Phrap-assembly tool and subsequently aligned using MEGA12: Molecular Evolutionary Genetics Analysis version 12 software (Kumar et al. 2024). Multiple sequence alignment was performed using the ClustalW method. Sequence similarity searches with sequences deposited in the National Center for Biotechnology Information (NCBI) database were conducted using the BLAST tool (//blast.ncbi.nlm.nih.gov/Blast.cgi). The best-fit nucleotide substitution model was selected using the Bayesian Information Criterion (BIC) implemented in MEGA12 (Kumar et al. 2024). Evolutionary history was inferred using the Maximum Likelihood method and the General Time Reversible model (Nei and Kumar 2000). Evolutionary analyses were conducted in MEGA12 (Kumar et al. 2024). Statistical analysis was performed using the bootstrap method with 1000 repetitions. *Parafilaroides* spp. (Dougherty 1946) was used as an outgroup taxon.

**Fig. 2:**
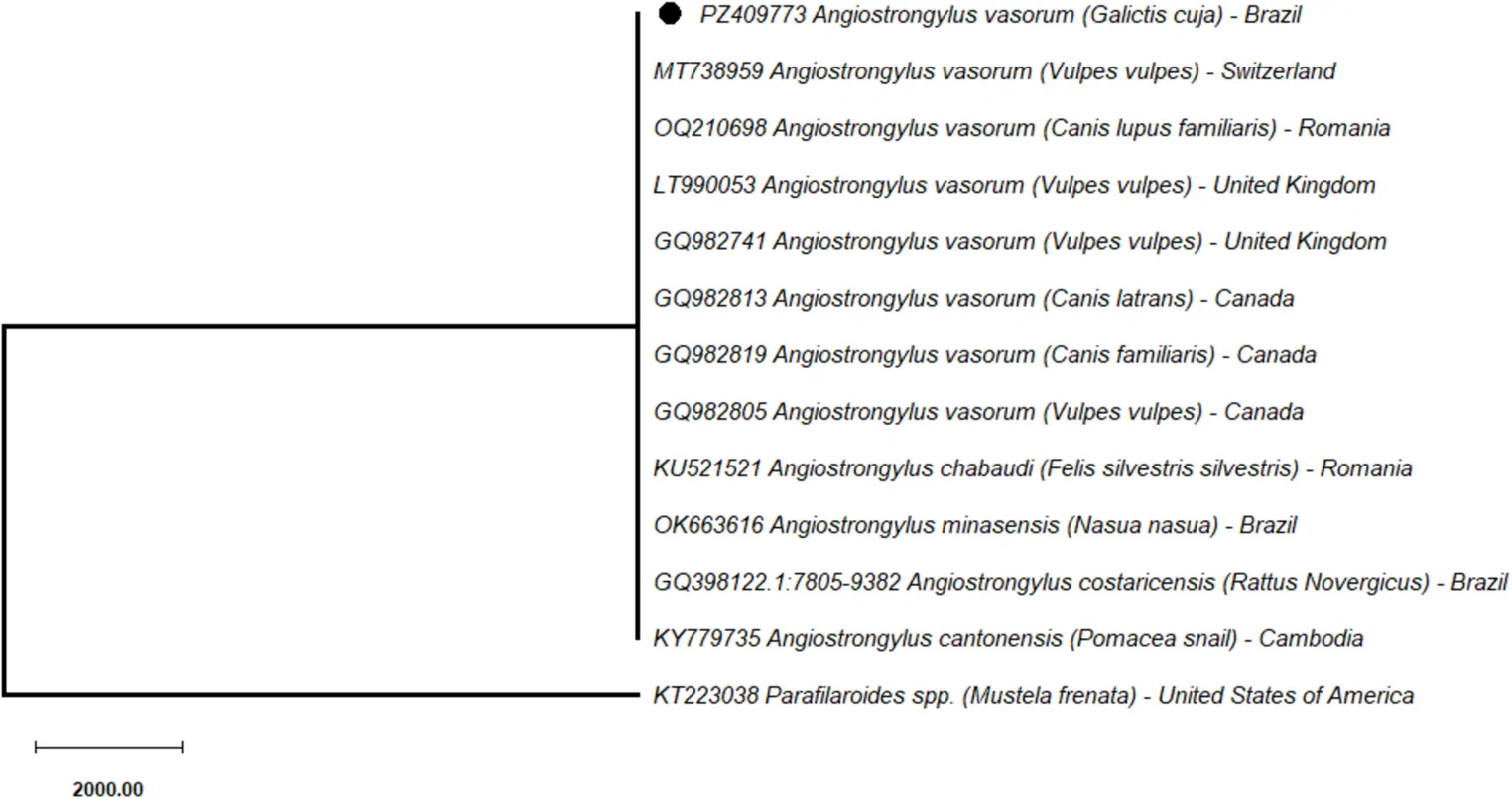
Phylogram showing phylogenetic relationships among *Angiostrongylus* species based on mitochondrial cytochrome *c* oxidase subunit I (*COX1*) gene sequences. GenBank accession numbers are shown before the taxon names. The scale bar represents the number of nucleotide substitutions per site, and branch lengths are proportional to the estimated evolutionary distances. The sequence obtained in the present study is indicated by a black circle. *Parafilaroides* spp. was used as the outgroup

Molecular analyses using PCR and genetic sequencing of the *COX1* gene confirmed the identification of *A. vasorum*, showing a similarity of 99–100% similarity to reference sequences of the same species used for comparison purposes and available in NCBI GenBank (GenBank accession numbers OQ210698, GQ982741, LT99053, MT738959, GQ982819, GQ982805 and GQ982813). Phylogenetic analysis further demonstrated that the obtained sequence clustered with European *A. vasorum* isolates (Fig. 2). The obtained gene sequence was deposited in the NCBI GenBank database under accession number PZ409773.

Anthelmintic treatment was performed with fenbendazole at a dose of 50 mg/kg for five days and no L_1_ larvae were detected in the follow-up fecal sample collected seven days after treatment.

## Discussion

Although the diagnosis of canine angiostrongylosis traditionally relies on the morphological identification of L_1_ larvae in fecal samples or bronchoalveolar lavage fluid, accurate species identification can be challenging and require experienced microscopists (Penagos-Tabares et al. 2018). Therefore, the combination of morphological characterization of larvae or adults with molecular techniques is important to ensure accurate parasite identification. This approach is particularly important when reporting infections in new host species, for which molecular confirmation is required to reliably establish the occurrence of the parasite. In this context, the *COX1* gene sequence obtained from the L_1_ larvae in the present study (GenBank accession number PZ409773) showed 99–100% similarity with *A. vasorum* detected in domestic dogs (*Canis lupus familiaris* Linnaeus 1758) from Romania (GenBank accession number OQ210698) and Canada (GenBank accession number GQ982819); red foxes (*Vulpes vulpes*) from United Kingdom (GenBank accession numbers GQ982741 and LT99053), from Switzerland (GenBank accession number MT738959) and from Canada (GenBank accession number GQ982805); and coyote (*Canis latrans* Say 1823) from Canada (GenBank accession number GQ982813). Furthermore, phylogenetic analysis demonstrated that the sequence obtained in this study clustered with European *A. vasorum* sequences used as references (GenBank accession numbers OQ210698, GQ982741, LT99053 and MT738959) (Fig. 2). In contrast, North American *A. vasorum* sequences grouped into a distinct clade (GenBank accession numbers GQ982819, GQ982805 and GQ982813). The obtained sequence remained clearly distinct from other *Angiostrongylus* species included in the analysis.

The evolutionary history of *A. vasorum* in South America remains unresolved. Based on analyses of mitochondrial COX1 and second ribosomal internal transcribed spacer (*ITS-2*) sequences, Jefferies et al. (2009) identified two distinct genotypes among European and Brazilian isolates and suggested that the occurrence of *A. vasorum* in South America may represent an ancient evolutionary event. The authors further proposed that South American populations could represent a cryptic species distinct from European isolates, indicating that the synonymization of *Angiostrongylus raillieti* Travassos 1972 with *A. vasorum* may have been premature. Subsequently, Lange et al. (2018) reported that Colombian isolates clustered with the European lineage based on *ITS-2* sequences, demonstrating that genetic affinity between South American and European isolates has also been observed using a different molecular marker. Similarly, the sequence obtained in the present study clustered with European *A. vasorum* isolates based on *COX1* analysis. More recently, Robleto-Quesada et al. (2024) reported that isolates from Costa Rica formed a distinct lineage from Brazilian and European isolates based on *COX1* sequences, suggesting the possibility of greater genetic diversity among American populations of *A. vasorum*. However, additional studies including a larger number of specimens, adult parasites, and complementary molecular markers are required to clarify the taxonomic significance of these findings. Together, these findings suggest that the genetic diversity and taxonomic relationships of *Angiostrongylus* populations in the Americas may be more complex than previously recognized. Therefore, the taxonomy of *Angiostrongylus* species reported in South America continues to be debated, and the taxonomic status of isolates from this region remains under discussion (Penagos-Tabares et al. 2018). In this context, additional molecular studies including a larger number of isolates and complementary genetic markers are needed to improve our understanding of the genetic diversity, taxonomy, and evolutionary history of *A. vasorum* in South America.

In Brazil, reports of *A. vasorum* have previously been described in domestic dogs (Gonçalves 1961; Giovannoni et al. 1985), the crab-eating fox (*Cerdocyon thous* Linnaeus 1766) (Vieira et al. 2017), the hoary fox (*Lycalopex vetulus* Lund 1842) (Lima et al. 1994), the ring-tailed coati (*Nasua nasua* Linnaeus 1766) (Vieira et al. 2008), and the tayra (*Eira barbara* Linnaeus 1758) (Vieira et al. 2008), including occurrences in the state of RS (Gonçalves 1961; Vivian 2015). However, this is the first report of *A. vasorum* parasitizing *G. cuja* in the country, expanding the number of known host species and reinforcing the presence of the parasite and the existence of a sylvatic cycle in the region more than ten years after the last record in the state (Vivian 2015).

*Angiostrongylus vasorum* has been reported in different regions worldwide, and its occurrence is closely associated with the distribution of its intermediate and paratenic hosts. The occurrence of *A. vasorum* in southern Brazil may be influenced by environmental conditions that favor gastropod populations, particularly in humid areas. In RS, recent climate changes, including the reduction in temperature range between seasons, may contribute to the establishment and persistence of suitable conditions for intermediate hosts. Furthermore, the low host specificity of *Angiostrongylus* species (de Almeida et al. 2020), along with the ecological characteristics of *G. cuja*, including its use of humid and forested habitats and its wide range of feeding habits (Yensen and Tarifa 2003), may increase opportunities for contact with infected hosts and contribute to parasite transmission.

In addition to climate, the international trade and movement of companion animals may contribute to the introduction and spread of *A. vasorum* into previously non-endemic areas. Dogs traveling between endemic and non-endemic regions may facilitate parasite dispersal, either by introducing the infection into new areas or by contributing to its establishment where suitable intermediate hosts are present. Furthermore, the reduction of forested areas has forced wild animals to migrate to new environments in search of anthropogenic food sources. These changes increase contact between wild and domestic animals, thereby contributing to parasite transmission and to the epidemiological changes currently observed in canine angiostrongylosis (Di Cesare and Traversa 2014).

Finally, previous studies have suggested that *A. vasorum* infections may be underdiagnosed due to nonspecific clinical manifestations and limitations associated with routine diagnostic procedures (Di Cesare and Traversa 2014). This hypothesis is plausible since the clinical signs of the disease are nonspecific and may be confused with other disorders (Colombo et al. 2021); larval shedding is intermittent (Rinaldi et al. 2014), which may compromise diagnostic sensitivity; and the detection of *A. vasorum* larvae in feces requires specific parasitological techniques (Di Cesare and Traversa 2014), such as the Baermann-Moraes method (Moraes 1948), which is still rarely employed in routine laboratory practice. In the present study, L_1_ larvae were detected by both the modified zinc sulfate centrifugal flotation and the Baermann-Moraes techniques. However, the Baermann-Moraes method recovered a substantially greater number of larvae (two vs. fifteen), facilitating both morphological identification and molecular characterization. Although flotation techniques using zinc sulfate may detect *A. vasorum* larvae (Schnyder et al. 2011; Di Cesare and Traversa 2014; Rinaldi et al. 2014), the Baermann method remains the preferred approach for larval recovery and diagnosis of canine angiostrongylosis (Di Cesare and Traversa 2014). Furthermore, considering that this helminth occurs in several countries across South America, infection by *A. vasorum* should be included among the differential diagnoses of cardiopulmonary diseases in canids. Therefore, veterinarians should consider *A. vasorum* in their clinical suspicions and incorporate specific diagnostic methods into routine laboratory investigations.

## Conclusion

Here, we report the first record of *A. vasorum* in *G. cuja* in Brazil. This finding expands the known host range of this nematode and provides further evidence of its ability to infect a diversity of wild carnivore species. Together with recent studies reporting increasing host and genetic diversity of *A. vasorum*, our findings reinforce the need for continued surveillance of wildlife hosts and contribute to a better understanding of the epidemiology and circulation of this parasite in South America. In addition, we provide a novel molecular sequence that may support future studies on the genetic diversity and phylogeography of *A. vasorum*.

## Data Availability

No datasets were generated or analysed during the current study.
